# The role of the practice nurse in the management of Postpartum depression

**DOI:** 10.1192/j.eurpsy.2022.665

**Published:** 2022-09-01

**Authors:** L. Hogea, C.D. Tabugan, A.-C. Bredicean, T. Anghel

**Affiliations:** 1 “Victor Babes” University of Medicine and Pharmacy, Department Of Neurosciences, Timisoara, Romania; 2 Psychiatric Clinic ” Eduard Pamfil” Timisoara, Neurosciences, timisoara, Romania; 3 University of Medicine and Pharmacy “Victor Babes” Timisoara, 5. neuroscience Department, Timisoara, Romania

**Keywords:** post-partum depression, nursing, management of post-partum depression

## Abstract

**Introduction:**

Nurses specializing in maternal and child health are poised to play a pivotal role in the early identification and prompt treatment of perinatal depression. Postpartum period it is well-known for presenting high-risk for the appearance of a mental illness.

**Objectives:**

This study has been carried out with the aim of investigating the level of knowledge of the nurses and their role in the management of post-partum depression.

**Methods:**

73 participants (n=73) were selected which are professional nurses. The data were collected through a questionnaire formed out of 16 questions. The questionnaire is structured on three parts: general information about the participants, the nurse’s knowledge about the postpartum depression, and the identification and the management of the patient’s cases.

**Results:**

73 of the nurses questioned, consider that they were not properly prepared for this role and they were not able to identify and manage the patients with post-partum depression. They also consider that the ideal training should contain more theoretical information. Amongst these (32, 87%) do not know the symptomatology, and 38, 35% are not aware of the risk factors of post-partum depression.

**Conclusions:**

Postpartum depression is seen in approximately 10% of women who have recently given birth, but also in 3, 3% of men. Despite of this numbers, the Romanian medical staff is not yet well prepared in facing this affection.

**Disclosure:**

No significant relationships.

